# Branched copolymer-stabilised nanoemulsions as new candidate oral drug delivery systems†

**DOI:** 10.1039/c8ra01944d

**Published:** 2018-04-09

**Authors:** James J. Hobson, Stephanie Edwards, Rebecca. A. Slater, Philip Martin, Andrew Owen, Steve P. Rannard

**Affiliations:** Department of Molecular and Clinical Pharmacology, University of Liverpool Block H, 70 Pembroke Place Liverpool L69 3GF UK aowen@liverpool.ac.uk; Department of Chemistry, University of Liverpool Crown Street Liverpool L69 7ZD UK srannard@liverpool.ac.uk

## Abstract

The delivery of drugs to the bloodstream *via* oral administration may suffer from a number of complications including poor dissolution, first pass metabolism and the active intervention of efflux transporters such as P-glycoproteins; drugs which are efflux substrates may cause considerable problems across many clinical conditions. Here we have employed a branch-polymer stabilised nanoemulsion strategy to create highly robust oil droplets (*e.g.* peanut oil, castor oil and soybean oil) containing different dissolved antiretroviral drugs used in the daily fight against HIV/AIDS. Although very limited difference in permeation through a Caco-2 gut epithelium model was seen for efavirenz, the permeation of the protease inhibitor lopinavir was considerably higher (approximately 10-fold) when applied to an epithelium monolayer in emulsion form than the control within an aqueous DMSO vehicle. The presented nanoemulsion approach may allow drug-specific permeation improvements for various drug substances.

## Abstract 2

Branched copolymers offer very efficient nanoemulsion stabilisers with benefits for oral drug delivery.

## Graphical abstract

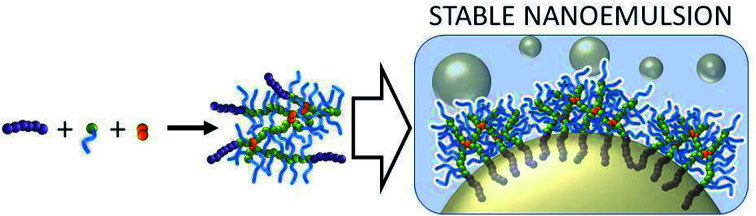

## Introduction

The continued growth of fundamental nanomedicine studies, and progression to clinical products, has allowed the improvement of a range of therapies, and the development of new treatment options, across a range of healthcare needs. These span psychiatric conditions^1^ to macular degeneration^2^ and immunosuppression;^3^ however, to date, the targeted delivery of cytotoxic drugs to cancer disease sites has taken priority.^4^ In contrast, chronic diseases are, in comparison, largely ignored by nanomedicine researchers. Several groups have reported exciting results of long-acting drug delivery from nano-enabled depot injections and prophylactic interventions to prevent disease.^4–6^ Importantly, oral delivery of therapies is the most patient-acceptable administration route, especially over long periods where the patient must self-administer; daily injection is therefore not relevant in these cases^7^ although long-acting parenterals/injectables are being increasingly sought to simplify dosing for chronic conditions.^8^

One such chronic disease is human immunodeficiency virus (HIV) infection and its progression to acquired immune deficiency syndrome (AIDS). HIV replication is suppressed by the daily co-administration of antiretroviral (ARV) drugs from multiple classes which simultaneously inhibit a number of targets within the viral replication cycle.^9^ HIV presents a major global public health threat. The UNAIDS 2016 global update estimates that (in 2015): 36.7 million people worldwide were living with HIV (range 34.0–39.8 million); there were up to 2.4 million new infections and up to 1.3 million AIDS-related deaths.^10^ The success of ARV therapies is observed as a considerable decline in mortality and morbidity^11^ and, subsequently, this leads to a growing demand for access to therapy and subsequent growth in the ongoing cost of global treatment. The combined UNAIDS 90 : 90 : 90 target^12^ of collectively establishing the HIV status for 90% of those infected, providing ARV therapy to 90% of people diagnosed with HIV, and achieving viral suppression for 90% of those receiving ARV therapy by 2020 will require novel solutions for drug delivery that optimises current manufacturing capacity. HIV requires lifelong daily dosing, often with large doses of drug combinations, leading to considerable risk of poor adherence to therapies and the potential for the emergence of drug resistance.^13^ For example, efavirenz (EFV) is a non-nucleoside reverse transcriptase inhibitor used in combination with tenofovir and emtricitabine as a once daily fixed-dose tablet (marketed as Atripla®) with a 600 mg/300 mg/200 mg drug ratio respectively.^14^ Low oral bioavailability contributes to the high doses and strategies that improve drug permeation from the gut to the blood may significantly reduce the administered dose and impact the per-patient cost of therapy. HIV patient groups were evaluated recently to establish their interest in novel depot injection options and a willingness to switch to nanomedicine alternatives was reported if relevant benefits can be shown.^15^

In recent years, the use of lipid-based nanoemulsions has been shown to offer benefits for drug delivery after topical,^16^ parenteral^16^ and oral administration. For drug administration *via* the gut, lipids have been reported to alter drug absorption through a combination of mechanisms including (a) enabling lymphatic uptake, (b) presenting poorly water-soluble drugs as pre-dissolved solutions, (c) stimulation of bile secretion that may facilitate absorption, and (d) inhibition of cellular mechanisms such as efflux transporters (*e.g.* P-glycoprotein; P-gp)^17^ or cytochrome P450 enzymes.^18^ Self-forming nanoemulsifying drug delivery systems (SNEDDS)^19^ have enhanced the bioavailability of a range of poorly-water soluble drug compounds, including very recent reports of a SNEDDS formulation of EFV.^20^ In addition, very recent reports have demonstrated solid self-nanoemulsifying oily formulation (S-SNEOF)^21,22^ approaches to enhance the delivery of the ARVs lopinavir (LPV) and darunavir, clinically well-established protease inhibitors. Self-emulsifying systems are isotropic mixtures that contain an oil phase, the drug compound and a combination of surfactants; they may also comprise a mixture of solvents, co-solvents, surfactant and drug. In these cases, determination of ternary or pseudo-ternary phase diagrams are important to identify mixtures that will spontaneously emulsify on contact with the gastrointestinal fluids. Co-surfactants (or co-solvents) may include 1-butanol, 1-propanol and ethanol and regions of the phase diagrams that offer potential for formulation may be limited. The use of a single emulsifier to generate nanoemulsions for oral drug delivery would greatly simplify the process of generating candidate nanomedicines and large scale manufacturing as the need to finely balance complex mixtures would be avoided.

Our own research has reported the use of branched amphiphilic polymers as extremely efficient emulsifiers. Branched pH-responsive copolymers were also shown to form large scale objects with remarkable stability and reversible hydrogen bonding between emulsion droplets.^23,24^ We hypothesised that analogous branched polymer-stabilised emulsions could be generated in the nanoscale to provide advantages for orally-dosed ARVs to enable HIV nanomedicine development. Here, we describe the controlled radical polymerisation synthesis of an amphiphilic branched copolymer able to stabilise nanoemulsions containing dissolved LPV or EFV; the tuning of nanoemulsion droplet sizes; and studies of cytotoxicity, drug permeation using a gut epithelium model and subsequent antiviral activity of the drug compounds showing their potential value as future candidate nanomedicines.

## Experimental

All experimental details, materials and methods are described in the ESI.†

## Results and discussion

### Branched copolymer emulsifier synthesis

In our previous reports of branched copolymer emulsifiers,^23,24^ we showed that the dodecanethiol chain transfer agent-mediated conventional free radical copolymerisation of oligo(ethyleneglycol) methacrylate (OEGMA, 1), methacrylic acid (MAA) and ethyleneglycol dimethacrylate (EGDMA, 2) generates relatively low molecular weight conjoined polymer structures with multiple hydrophobic chain ends. The multiple dodecyl chain ends were able to strongly interact with the oil-phase of dodecane and to form highly stable emulsion droplets in water in the absence of additional surfactants or polymers. Linear polymer structures, synthesised in the absence of EGDMA, were poor stabilisers in comparison.

**Scheme 1:**
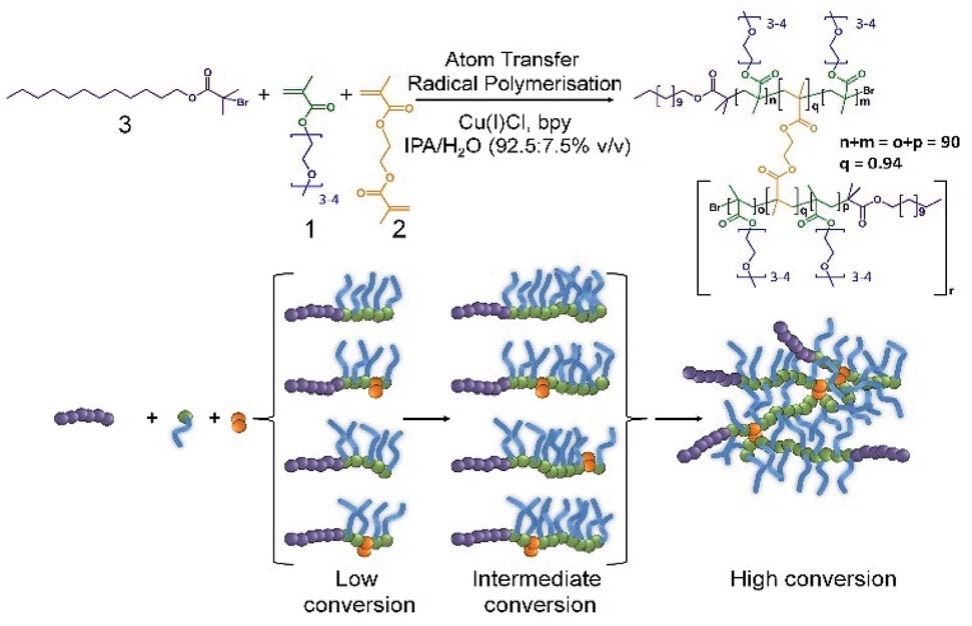
Synthesis of the branched copolymer emulsifier employing copper-catalysed ambient temperature ATRP of oligoethylene glycol monomethacrylate and ethylene glycol dimethacrylate in IPA/water. A dodecylbromoisobutyrate initiator was employed with a Cu(i)Cl/Bpy catalytic system (1/2) at an overall concentration of 44 v/v%.

Our subsequent research has also shown that the formation of polymer nanoparticles from the controlled radical branched amphiphilic block copolymerisation of OEGMA, *n*-butyl methacrylate and EGDMA yielded very high molecular weight materials, under atom transfer radical polymerisation (ATRP) conditions, and shaped nanoparticles could be directly synthesised through also incorporating multifunctional initiators.^25,26^ Here, we have combined these approaches to generate high molecular weight branched hydrophilic copolymers of OEGMA (approximately 300 g mol^−1^) and EGDMA using the hydrophobic ATRP initiator dodecyl 2-bromoisobutyrate (DodBiB, 3) under copper catalysed conditions using a 92.5 : 7.5% v/v IPA/water solvent mixture (Scheme 1).

Comparative studies of the linear and branched DodBiB-initiated copolymerisation of OEGMA and OEGMA/EGDMA respectively, targeting poly(OEGMA) chains with a number average degree of polymerisation (DP_*n*_) of 90 units, showed nearly identical kinetics, reaching approximately 100% monomer conversion between 7–8 hours at ambient temperature (Fig. S1†); near-linear semi-logarithmic plots were also observed over this time showing no observable difference in the progression of the polymerisation in the presence or absence of EGDMA, as seen with several previous branched vinyl polymerisations conducted under ATRP conditions. Branched copolymer solutions were studied up to concentrations of 10% (w/v), and all appeared visually transparent with subsequent measurement by dynamic light scattering (DLS) showing very weak scattering and no substantial self-assembly.

Despite the near identical polymerisation kinetics, the presence of EGDMA within the branched copolymerisation (0.95 : 1 brancher to initiator ratio) yielded an amphiphilic copolymer, poly(OEGMA_90_-*co*-EGDMA_0.95_), with a number average molecular weight (*M*_n_) of 218 330 g mol^−1^ and weight average molecular weight (*M*_w_) of 3 181 000 g mol^−1^ (Fig. S2†). The EGDMA-derived branching between poly(OEGMA)_90_ chains, therefore, yields a polymer architecture comprising a dense poly(OEGMA) region with multiple hydrophobic dodecyl chain ends. From a number average perspective, therefore, the most abundant species within the polymer sample has approximately 8 conjoined highly hydrophilic polymer chains, each with a dodecyl hydrophobic chain end; from a weight average perspective, the polymer structures contributing at least half of the mass of the polymer sample have at least 85 nominal conjoined chains with their corresponding dodecyl chain ends. This is an important consideration as the polymer is used as an emulsion stabiliser as a weight% ratio with respect to the added oil and water phases.

### Selection of oil-phase

Selection of the oil-phase for the targeted nanoemulsions was made based on several criteria. As the targeted administration route is *via* oral dosing, we selected oils that were either commonly used in foods or pharmaceutical products, were present on the FDA Generally Recognised as Safe (GRAS) or FDA/CDER Inactive Ingredients lists or were designated as Generally Recognised as Safe and Effective (GRASE).

**Fig. 1:**
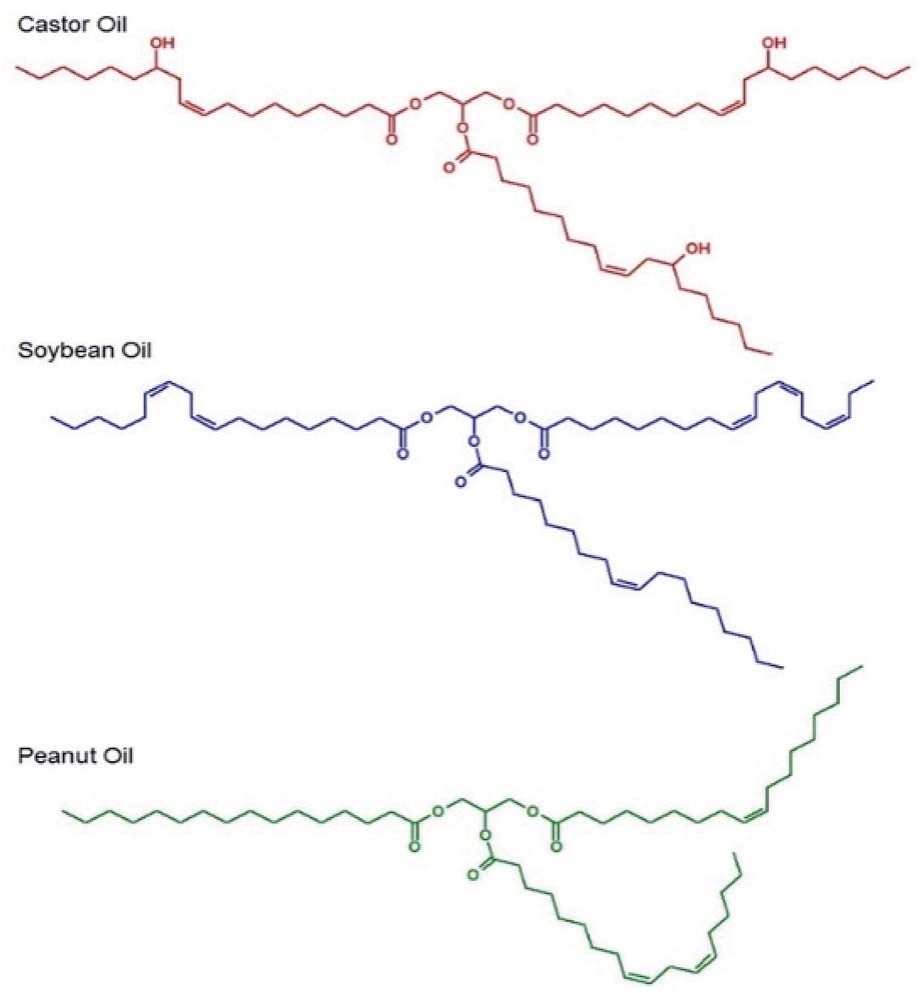
Chemical structures of the natural oils studied for nanoemulsion formation.

Importantly, the GRAS list relates to food additives, the Inactive Ingredients list describes excipients used in previously FDA-approved medicines and the GRASE designation is reserved for well-established and traditional drug substances that do not require new regulatory approval. The oil-phase also needed to be a good solvent for the drug substances chosen, namely LPV and EFV, and be capable of stable emulsion formation; as such, chemical diversity within the oils was important. Three candidate oils namely castor oil,^27^ soybean oil^28^ and peanut oil^29^ (Fig. 1) were selected for initial consideration and were subjected to drug solubility and unloaded nanoemulsion formation studies prior to detailed analysis of drug-loaded nanoemulsions.

Drug solubility in these oils was measured as ≥50 mg mL^−1^ for EFV in castor oil, soybean oil and peanut oil and approximately 25 mg mL^−1^ in castor oil and <12 mg mL^−1^ in soybean oil and peanut oil for LPV.

### Branched copolymer stabilised nanoemulsions

**Fig. 2:**
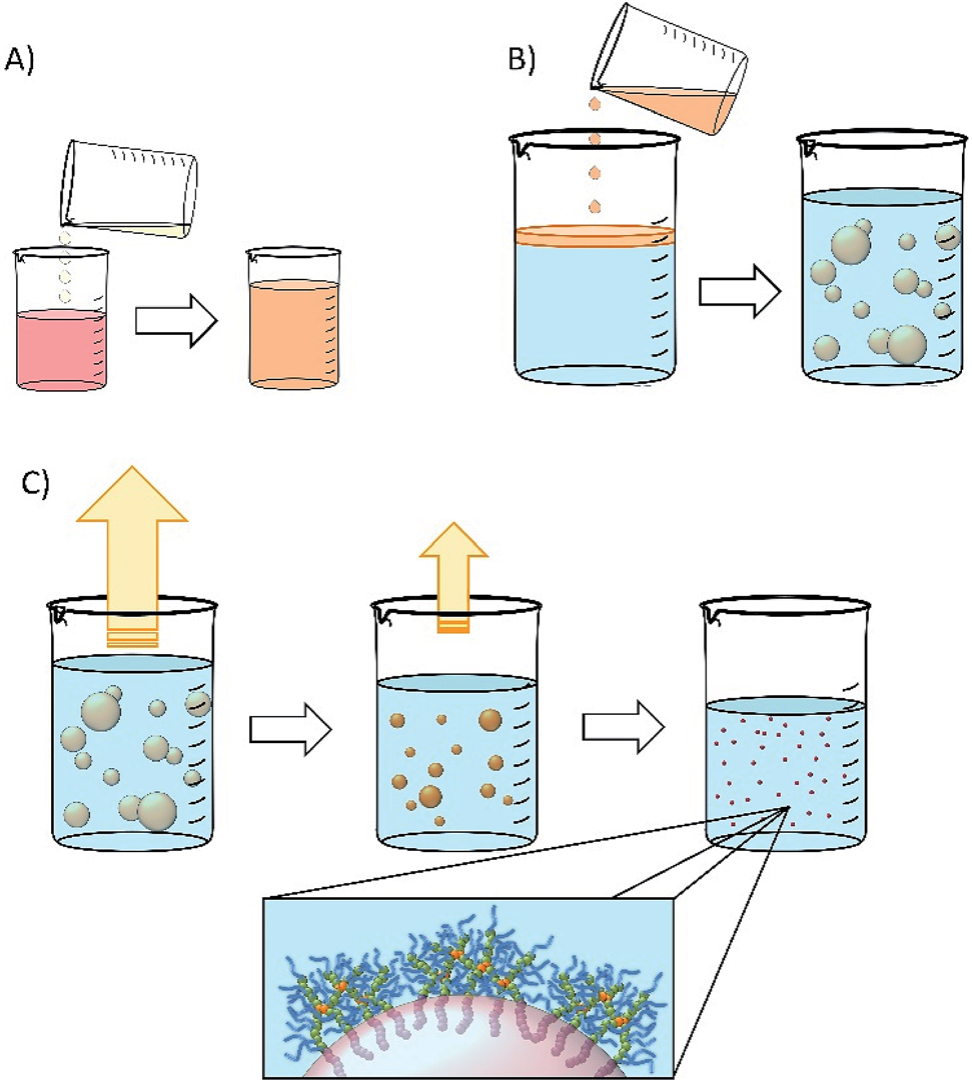
Schematic representation of nanoemulsion formation. (A) Natural oil solution of drug is diluted with volatile oil component (water-immiscible solvent); (B) drug solution in mixed volatile/non-volatile oils is added to an aqueous solution of branched copolymer stabiliser (typically 5% w/v) and emulsified; (C) the volatile component of the dispersed phase is removed by evaporation leading to a decrease in the oil droplet volume and diameter.

Nanoemulsions may be generated using a range of techniques^30–33^ and we chose to adopt a low-energy co-solvent evaporation strategy to prevent any impact of the processing on the drugs that were ultimately utilised. This strategy employs an admixture of volatile and non-volatile oils to comprise the dispersed phase (Fig. 2A) that is homogenised into an aqueous continuous phase containing the dissolved branched copolymer stabiliser (Fig. 2B); no additional stabilisers are present. Removal of the volatile oil from the resulting emulsion by evaporation leads to shrinkage of the oil droplets (Fig. 2C) and final droplet size is controlled by the relative volumes of the volatile and non-volatile oils.

Ethyl acetate was selected as the volatile oil (co-solvent) component of the nanoemulsion formation process as it is listed on the FDA/CDER list of Inactive Ingredients as being present in intramuscular, oral, topical and transdermal products; it is also a Class 3 solvent listed within the European Medicines Agency's International Council for Harmonisation of Technical Requirements for Pharmaceuticals for Human Use guidelines for residual solvents, establishing this solvent as having a low human toxicity potential and no requirement for a health-based exposure limit.

**Fig. 3:**
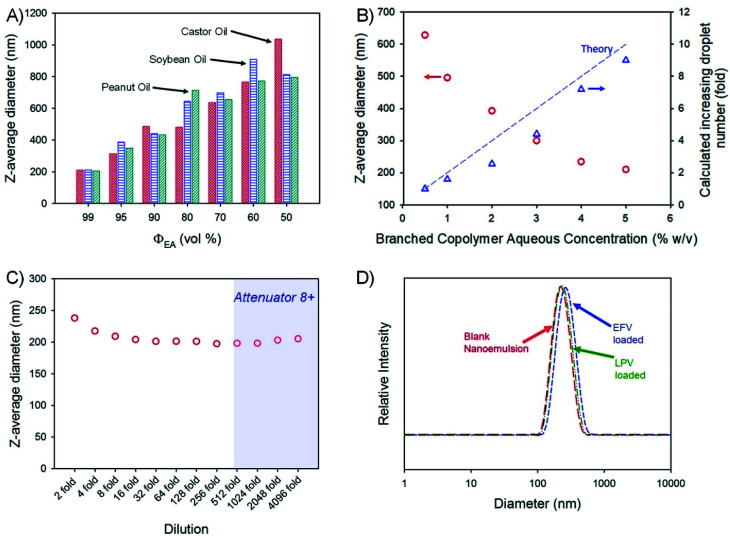
Analysis of branched copolymer stabilised nanoemulsions. (A) *z*-average diameter (dynamic light scattering) variation of nanoemulsions of castor oil (red), soybean oil (blue) and peanut oil (green) after removal of varying amounts of ethyl acetate as the volatile component of the dispersed phase; (B) variation of oil droplet size after volatile component removal for castor oil emulsions generated using different concentrations of branched copolymer emulsifier in the aqueous phase (red circles) and a comparison of corresponding calculation of increasing droplet number derived from measured droplet diameters and the mass of oil present (blue triangles); (C) determination of castor oil nanoemulsion stability to dilution (dynamic light scattering) – shaded zone shows data which may be unreliable; (D) castor oil nanoemulsion droplet size distributions (dynamic light scattering) of either blank (red) or loaded with either efavirenz (blue) or lopinavir (green).

Drug-free emulsions were initially formed using a 1 : 1 ratio of aqueous and oil-phases (3 mL of each) followed by homogenisation using a hand-held Ultra-Turrax T-25; the aqueous phase contained the amphiphilic branched polymer emulsifier at 5% (w/v). The volume fraction of ethyl acetate (*Φ*_EA_) within the combined oil phase was varied from 99 vol% through to 50 vol% for each non-volatile oil-phase, leading to varying emulsion droplet *z*-average diameters (*D*_*z*_) ranging from 205 nm (peanut oil; *Φ*_EA_ = 99 vol%) to 1.04 μm (castor oil; *Φ*_EA_ = 50 vol%), as measured by DLS (Fig. 3A). Given the highest drug solubilities being achieved for castor oil, a more detailed analysis of drug-loaded nanoemulsion formation using *Φ*_EA_ = 99 vol% was conducted with this non-volatile oil phase.

Variation of branched copolymer concentration within the aqueous phase of the emulsion (castor oil; *Φ*_EA_ = 99 vol%) from 0.5% to 5% (w/v) led to a systematic decrease in the final nanoemulsion droplet size from 630 nm to 210 nm (Fig. 3B) as would be expected for classical emulsifier behaviour; as the volume of non-volatile oil is identical in these experiments, assuming the interfacial surface area is constant and entirely stabilised by the branched copolymer and accepting the limitations of DLS measurement, the expected increase in droplet number required to conserve the interfacial surface area correlates well with the increasing copolymer concentration (Fig. 3B), suggesting a low concentration of free copolymer is present within the final nanoemulsions. Ongoing detailed analysis is being undertaken to establish this in a quantitative manner.

Orally-dosed emulsions are expected to be subject to considerable dilution, therefore, the robustness of the blank (no drug-loading) 210 nm (castor oil; *Φ*_EA_ = 99 vol%) sample to serial dilution was studied (Fig. 3C). At an initial relatively high concentration, the observed *D*_*z*_ values were slightly above the values determined under our standard conditions (100-fold dilution to obtain an attenuator value = 5) but decreased to a near stable value up to 4096-fold dilution; *D*_*z*_ measured above a 512-fold dilution should be treated with some caution as the automatically set attenuator for the DLS was reaching very high values. The specific differences in the observed *D*_*z*_ values (presented in Fig. 3C) are not significant as highlighted by the lack of meaningful changes in the breadth or peaks of the particle size distributions across the dilution range (Fig. S3†). The observed resistance to dilution implies a surface active behaviour more akin to particle stabilised emulsions rather than traditional polymer and copolymer surfactant systems that would be expected to pass through a critical micelle concentration with subsequent emulsion destabilisation. Interestingly, the stability of this nanoemulsion was also studied by storing the sample under ambient conditions for 2 years with no observable change in droplet size or emulsion destabilisation during this time (Fig. S4†). Due to these highly positive results, no iteration of polymer structure was conducted.

The impact of loading EFV or LPV into the castor oil on nanoemulsion formation was monitored and no appreciable change in the polydispersity of the sample or the *D*_*z*_ values was seen (Fig. 3D) with only a slight increase in the distribution for EFV-containing samples loaded at the higher drug concentration of 50 mg mL^−1^. The zeta potential (*ζ*) values for the blank and drug-loaded nanoemulsions were all measured to be neutral (*ζ* < −1 mV) as expected from a highly branched OEGMA containing copolymer.

### Characterisation of branched copolymer and nanoemulsion cytotoxicity

Two relevant cell types were studied to investigate and rule out overt cytotoxicity from the potential future oral dosing of the branched copolymer stabilised nanoemulsions. The Caco-2 cell line is derived from human epithelial colorectal adenocarcinoma cells and used as a model of the human gut epithelium which the nanoemulsions would come into intimate contact with after administration. If appreciable gut absorption is observed, and a portion of the nanoemulsion dose enters the hepatic portal vein it will be transported directly to the liver, hence cytotoxicity of HepG2 cells, a liver carcinoma cell line, was also evaluated; the implications for potential lymphatic uptake was not evaluated. The inherent cytotoxicity of the branched copolymer was evaluated using the CellTiterGlo® ATP assay whilst the stabilised nanoemulsions were studied using a 3-(4,5-dimethylthiazol-2-yl)-2,5-diphenyltetrazolium bromide (MTT) assay which employs the reduction of MTT to formazan to establish changes in mitochondrial enzyme activity (Fig. S5†). The branched copolymer studies yielded half-maximal cytotoxic concentration (CC_50_) values of 0.98% w/v and 2.9% w/v against HepG2 and Caco-2 cells respectively indicating very low cytotoxicity against these cell types. When assessing the unloaded and drug-loaded nanoemulsions against Caco-2 cells, and comparing to aqueous/DMSO solutions of the drug compounds, the EFV drug control exhibited an CC_50_ value = 55 μM but all other tests showed no overt cytotoxicity over the concentration ranges tested, demonstrated by the non-convergence of sigmoidal dose response curves (Fig. S6†). Against HepG2 cells, the control aqueous/DMSO drug solutions and all blank or loaded nanoemulsions displayed no observable cytotoxicity across the concentration ranges tested (Fig. S7†).

### Model transcellular permeation studies using Caco-2 epithelial monolayers

The apparent permeability (*P*_app_) of the candidate drug-loaded nanoemulsions were determined using the Caco-2 cells after forming a confluent monolayer, with tight junctions between the cells (confirmed by TEER), on a porous transwell membrane; this is a widely-used model for oral absorption.^34^ Within this assay, the cells polarise to present microvilli on the gut (apical; A) side of the cells,^35^ reproducing the morphology and function of the small intestine. The blood (basolateral; B) side of the cells models the interface with the systemic circulation.

The apical to basolateral (A → B) *P*_app_ of the nanoemulsion containing EFV showed no significant difference in gut to blood permeation after 1 hour but a significantly higher *P*_app_ after 2 hours (*p* ≤ 0.05) (Table 1).

**Table 1:** Apparent permeation of efavirenz and lopinavir through Caco-2 monolayers when applied as either an aqueous DMSO solution or an aqueous nanoemulsion in castor oil. 2 hour data shown and all samples with the exception of nanoemulsion LPV satisfied sink conditions due to rapid permeation

	A → B (± SD)	*p* value	B → A (± SD)	*p* value	B → A/A → B
**EFV**					
Aq/DMSO	8.21 × 10^−6^ (1.04 × 10^−6^)	≤0.05	2.55 × 10^−5^ (1.70 × 10^−6^)	≤0.05	3.11
Nanoemulsion	1.11 × 10^−5^ (1.73 × 10^−6^)		1.23 × 10^−5^ (1.59 × 10^−6^)		1.10

**LPV**					
Aq/DMSO	2.70 × 10^−6^ (1.93 × 10^−6^)	≤0.05	1.29 × 10^−5^ (4.15 × 10^−6^)	≥0.05	4.78
Nanoemulsion	6.37 × 10^−5^ (1.12 × 10^−5^)		6.02 × 10^−6^ (4.63 × 10^−6^)		0.09

The nanoemulsion conferred a considerable impact upon the behaviour of LPV, where almost an order of magnitude higher A → B *P*_app_ (*p* ≤ 0.05) was seen after 2 hours of the experiment compared to the aq/DMSO drug solution control. This is a highly interesting result as LPV is very poorly-absorbed *in vivo* and is a known substrate for a range of drug transporters found within the intestine and modelled in Caco-2 cells;^36^ efflux transporters, such as P-gp,^37^ actively pump drugs back into the gut (often against a concentration gradient), negatively impacting bioavailability. The nanoemulsion appears to enable the passage of LPV through the epithelial barrier whilst effectively hiding the drug from, or inhibiting, a range of potential mechanisms acting on the free drug compound.

It is clearly important to study permeation in the apical to basolateral (A → B) direction, which models absorption from the gut to blood, but *P*_app_ in the basolateral to apical (B → A) direction is also critical to understand as the balance of B → A/A → B permeation provides a more accurate determinant of predicted bioavailability. The EFV-loaded nanoemulsions exhibited significantly lower B → A permeation than the aq/DMSO drug solution for both 1 hour (Table S1; Fig. S8†) and 2 hour time points whilst the LPV-loaded nanoemulsions showed higher B → A permeation in the first hour and no significant difference to the aq/DMSO drug solution in the second hour (Table S1; Fig. S9†). Despite these differences, the calculated B → A/A → B ratios for the LPV-nanoemulsion are approximately 50% of the aq/DMSO solution in the first hour and approximately 2% of the aq/DMSO solution at the 2 hour timepoint; for EFV nanoemulsions, the B → A/A → B ratio is approximately 20% of the aq/DMSO solution after 1 hour and approximately 35% after 2 hours (Table S1†). The impact of the nanoemulsion in mediating the permeation of both drugs in both directions, and the ability to dramatically affect the B → A/A → B ratio is therefore clear but much more pronounced for LPV. This is highly encouraging, but it is important to note that this model is a static representation of the gut epithelium^38^ and *in vivo* experimentation is required to confirm the benefit in a more realistic environment.

### Antiviral activity of drug-loaded nanoemulsions against HIV-1 IIIB

The potential ability to enhance the oral permeability of the antiretroviral drugs LPV and EFV may lead to increased drug absorption and a subsequent decrease in oral dose required to treat HIV patients. Drug permeation through the gut model employed here provides no information about whether free drug or intact nanoemulsions have traversed the epithelium. The potential for nanoemulsions entering the systemic circulation after *in vivo* absorption necessitates assessment of antiviral activity of the ARV-containing droplets and this was undertaken using HIV-1 IIIB-infected MT4 cells. EFV and LPV intervene at different points within the replication cycle with EFV inhibiting reverse transcription of the viral RNA, and LPV inhibiting protease-mediated maturation of virions. Importantly, the experiment conducted here is in the presence of cells with ongoing viral replication, therefore at least for EFV the drug must enter the cell to show any inhibition of further replication. Activity against the virus was determined by using an MTT cell viability assay to assess the number of live cells as viral replication leads to cell death.

**Fig. 4:**
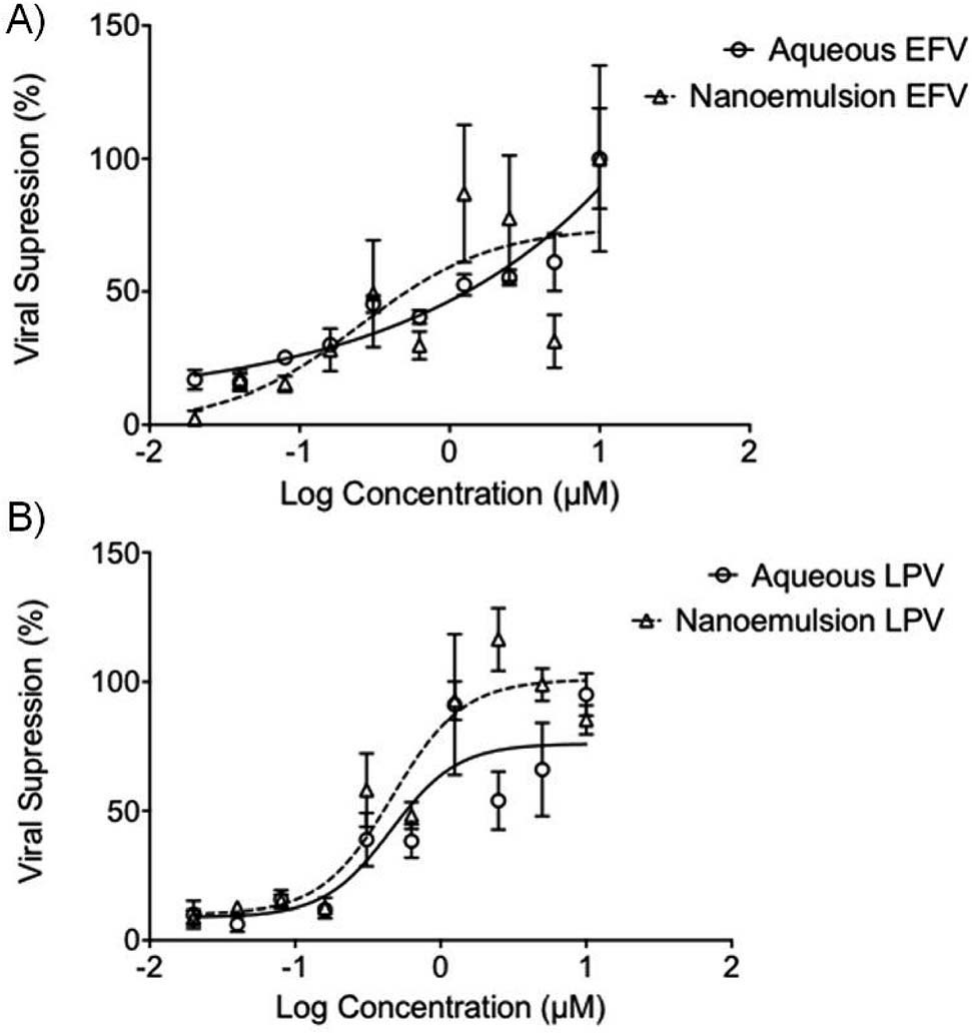
Antiviral activity of aqueous (circles) and nanoemulsion (triangles) formulations containing (A) EFV; and (B) LPV after 5 days incubation with MT4 cells and HIV-1 IIIB. Viral kill was derived by using MTT assay to quantify the metabolic activity of the MT4 cells, using this a proxy of cellular viability. Increased viability indicated an increased viral kill, as compared to control untreated MT4 cells, exposed only to HIV-1 IIIB. Data expressed as ± SEM, *N* = 4.

Interestingly, the antiviral activity of the aq/DMSO EFV solution (approximately 1.25 μM) was very similar to the EFV-loaded nanoemulsion with an IC_50_ value of 0.25 μM (Fig. 4A); both the aq/DMSO LPV solution and LPV-loaded nanoemulsion were near-identical at IC_50_ = 0.46 μM (2 significant figures) (Fig. 4B). Unloaded nanoemulsion controls showed no inherent antiviral activity against HIV-1 IIIB as expected. The ability of the nanoemulsions to provide equivalent antiviral activity to the aq/DMSO solutions mitigates concerns about a loss of efficacy if intact nanoemulsion droplets are absorbed.

## Conclusions

Nanoemulsions offer the potential to dissolve poorly-water soluble drugs within oils that are typically used in food products and overcome the poor gut absorption that is often associated with such compounds. A successful oral drug delivery system based on nanoemulsions needs to exhibit a range of important factors including: (1) ready and reproducible emulsion formation; (2) stability on storage; (3) significant drug loading; (4) low toxicity; (5) retention of drug activity (in emulsion and released drug formats); and (6) facilitation of drug passage across natural barriers. Here we have demonstrated much of what would be required to justify significant additional research as the system that was designed and studied has accomplished not only the above criteria but an additional benefit is the resistance to dilution as this not only offers stability within the body after administration (if absorbed intact) but also the potential for ready dose adjustment for individual patients, especially in a paediatric setting where there is limited availability to appropriate dosage forms.^39–41^

Several aspects of this candidate therapeutic option are yet unstudied, including whether such benefits can be achieved *in vivo*, and whether the nanoemulsions traverse the gut model (or real gut epithelium) as intact droplets. If this is the case, the possibility of generating circulating nanoemulsion droplets after oral administration provides further bespoke opportunities for future research. Mechanistically, the exact route that the drug has taken to traverse the epithelium is unclear. One possibility is that the surrounding branched polymer presents a considerable amount of bound water and a near-neutral *ζ* that the nanoemulsion does not invoke any direct efflux response by the cells and, by encapsulating the drugs within the oil phase, they may be effectively smuggled across the membrane. Other possibilities include localised high concentrations of drug being released from the oil-phase or paracellular diffusion (between cells) of the nanoemulsion droplets. The drug-specific nature of the benefits seen for LPV, over the EFV comparative nanoemulsions, does suggest that this may be the overcoming of drug-related constraints but this is yet to be confirmed.

Further work is needed to confirm the biocompatibility and safety of this nanoemulsion system, to understand the mechanistic aspects of the observed benefits and to establish *in vivo* confirmation of the *in vitro* studies presented here.

## Conflicts of interest

JJH, SE, AO and SR are co-inventors of patents related to emulsion and nanoemulsion drug delivery.

## Supplementary Material

RA-008-C8RA01944D-s001
