# Supplementary material for: Branched copolymer-stabilised nanoemulsions as new candidate oral drug delivery systems
Source: RSC Adv. 2018 Apr 9;8(23):12984–91. doi: 10.1039/c8ra01944d (PMC9079742; doi:10.1039/c8ra01944d)
Supplement: RA-008-C8RA01944D-s001 [file RA-008-C8RA01944D-s001.pdf]

Electronic Supplementary Material (ESI) for RSC Advances.
This journal is © The Royal Society of Chemistry 2018

Branched polymer-stabilised nanoemulsions as new candidate oral drug delivery systems.

James J. Hobson,[a] Stephanie Edwards,[b] Rebecca. A. Slater,[b] Philip Martin,[b] Andrew Owen[b,*] and Steve P. Rannard[b,*]

[a] Department of Chemistry, University of Liverpool, Crown Street, Liverpool, L69 7ZD, UK
[b] Department of Molecular and Clinical Pharmacology, University of Liverpool, Pembroke Place, Liverpool, L69 3GF, UK

Email: aowen@liverpool.ac.uk; srannard@liverpool.ac.uk

Supplementary Information

Experimental Details

Materials

Oligo ethylene glycol monomethyl ether methacrylate (OEGMA 300 g/mol), 2-dodecylbromoisobutyrate (DodBiB), tetrahydrofuran (THF), ethylene glycol dimethacrylate (EGDMA), copper chloride, 2,2'-bipyridine (BPY), anisole, methanol, isopropyl alcohol, Dowex Marathon MSC acid form beads, aluminium oxide, deuterated methanol (MeOD), acetone, castor oil, peanut oil, soy bean oil, sesame oil, Dulbecco's modified Eagles medium (DMEM), Roswell Park Memorial Institute 1600 medium (RPMI 1600), Hank's balanced saline solution (HBSS), Trypsin-EDTA, (3-(4,5-dimethylthiazol-2-yl)-2,5-diphenyltetrazolium bromide (MTT), glacial acetic acid and dimethylformamide (DMF) were all purchased from Sigma-Aldrich (Dorset, UK). Oxygen-free Nitrogen gas was supplied by BOC Industrial gases (Guildford, UK) and NMR tubes were supplied by Bruker UK (Coventry, UK). Fetal bovine serum was supplied by Life Technologies (Paisley, UK), cell culture flasks, cell culture plates, 10 and 25 mL pipettes, pipette tips and isopropyl alcohol were all purchased from Fisher Scientific (Loughborough, UK). Cell TiterGlo® ATP assay was supplied by Promega (Southampton, UK). Caco-2 human epithelial colorectal adenocarcinoma cells and HepG2 hepatocellular carcinoma cells were both purchased from the American Tissue Culture Collection (ATCC) (Manassas, US). 24 well HTS® Transwell plates were supplied by Corning Life Sciences (Amsterdam, The Netherlands). HIV-1 IIIB was acquired from the National Institute of Biological Standards and Control (Potters Bar, UK) and MT4 human T-Cell leukaemia cell line was purchased from Public Health England General Cell Collection (Porton Down, UK).

Characterisation

Triple detection size exclusion chromatography (SEC) was performed using Malvern Viscotek instrument equipped with a GPCmax VE2001 auto-sampler, two Viscotek T6000 columns (and a guard column), a refractive index (RI) detector VE3580 and a 270 Dual Detector (light scattering and viscometer) with a mobile phase of THF at a flow-rate of 1 mL min^{-1}. Dynamic Light Scattering (DLS) was used to determine droplet diameter and surface charge using a Malvern ZetaSizer Nano ZS instrument. Samples were measured in plastic zeta cells, which allowed for measurement of both z-average diameter and ζ-potential. Samples were diluted to obtain a laser attenuation of 5 or 6, and measured 3 times for both diameter and surface charge, with each measurement having an automated number of scans, as determined by the Zetasizer software. The average value from the 3 scans was reported and the temperature within the measurement cell was set to a constant 25 ºC.

Atom transfer radical polymerisation of poly($OEGMA_{90}$-co-$EGDMA_{0.95}$)

In a typical experiment, dodecylbromoisobutyrate initiator (0.118 g, 3.52 x10^{-4} moles), OEGMA, MW=300 g/mol (targeted DP_n = 90) (9.44 g, 3.15 x 10^{-2} moles) and EGDMA brancher (62.5 µL, 3.3 x10^{-4} moles) were weighed into a round bottom flask. The flask was equipped with magnetic stirrer bar, sealed and degassed by bubbling with N_2 for 20 minutes and maintained under N_2 at ambient temperature. IPA:H_20; 92.5:7.5 (12 mL) was degassed separately and subsequently added to the monomer/initiator mixture. The catalytic system; Cu(I)Cl (0.034 g 3.52 x10^{-4} moles) and BPY (0.137 g, 8.8 x10^{-4} moles), were added under a positive nitrogen flow in order to initiate the reaction. The polymerisations were stopped after 8 hours, when conversions had reached over 98 % determined by ^{1}H NMR using the vinyl CH_2 peaks and protons of the polymer backbone. The polymerisation was stopped by diluting with a large excess of THF, which caused a colour change from dark brown to a bright green colour. Residual solvent was removed from polymer batches using rotary evaporation, with vacuum set to 200 mBar and temperature of the water bath at 55°C. Cold tap water was pumped around the condensing vessel and solvent collected in a Wolffe bottle attached to the bottom of the condenser. Polymer purification was carried out by firstly dissolving the polymer in the minimum volume of THF and adding dropwise to a conical flask containing ice-cold petroleum ether 60-80 in which it precipitated out of solution. To maintain the low temperatures, the conical flask was placed in a crystallisation dish and surrounded by dry ice. After addition of all polymer solution the petroleum

ether was decanted and polymer sample dried using rotary evaporation, to remove residual organic solvent.

Nanoemulsion synthesis

Firstly, non-volatile oil and volatile co-solvent were mixed in a desired ratio. A range of ratios (99:1 volatile co-solvent to non-volatile oil, up to 50:50 volatile co-solvent to non volatile oil) was examined in order to find the optimum ratio based on size of nanoemulsion droplets. At 99:1 nanoemulsion droplets were below 300 nm in diameter. Thus 2.970 mL of volatile co-solvent (ethyl acetate) was added to 30 μL of non-volatile oil (castor oil), to make a total oil/co-solvent phase of 3.00 mL. To this, 3 mL of a 5% w/v concentration of polymer dissolved in water was added and this two-phase mixture was homogenised for 2 minutes using an Ultra Turaxx T-25 digital homogeniser fitted with an S 25 N - 10G dispersing element, and set to maximum speed of 25,000 RPM. During the 2 minutes, the vial was rotated clockwise for 30 seconds, anticlockwise for 30 seconds and then up and down for 1 minute, to achieve a creamy emulsion. After homogenisation, the co-solvent was evaporated over a period of 24 hours by removing the vial cap and leaving in a fume hood at ambient temperature.

Cellular Cytotoxicity Assay

Approximately 100,000 cells per 100 μL were seeded per well in a 96 well tissue culture plate, leaving 24 hours for cells to adhere to the plate. After this 24-hour period, media was aspirated from all wells and replaced with fresh complete culture media containing appropriate sample concentrations. A maximal drug concentration of 10 μM was used, decreasing in a 1:1 dilution series. For blank emulsion samples were an equivalent volume of nanoemulsion was used to prepare the sample, such that the volume of nanoemulsion was the same as those in the drug loaded nanoemulsion samples. Plates were left for between 1 and 5 days, after which time 20 μL of 5 mg/mL MTT reagent (3-(4,5-dimethylthiazol-2-yl)-2,5-diphenyltetrazolium bromide) was added to every well and left for 2 hours. At the end of the 2-hour incubation, 100 μL of MTT lysis buffer (50% DMF, 10% SDS, adjusted to pH 3.2 using glacial acetic acid) was added to each well and left for a further 24 hours in order to dissolve formazan crystals. Plates were then read on a Tecan GENios plate reader (Tecan Group, Manndorf, Switzerland) reader set to absorbance mode, with wavelength at 560 nm.

For Cell TiterGlo® Assay, 100 μL of polymer sample was added to each well for a dilution 1:1 series with a maximal of 5% (w/v) and left for 5 days incubation. 180 μL was removed from each well and 20 μL of ATP reagent was added, leaving each well with a volume of 40 μL. The ATP reagent lysed the cells, and reacted with the ATP of the cells to produce a luminescent signal. After a 10-minute incubation to stabilise the signal, the plates were read using a TECAN GENios plate reader, set to luminescence mode, with 2 seconds of shaking prior to reading.

Caco-2 Transcellular Permeation Model

Caco-2 cells were seeded at a density of 35,000 cells per well in the apical chamber of a 24 well Corning® HTS® transwell plate, and left to adhere for 24 hours. After this time, culture media was replenished by removal with an aspirator, also removing those cells that had not adhered. Culture media used was DMEM supplemented with 15% FBS, which was continually replenished every other day for a 3-week period. After three weeks had passed, the formation of an intact caco-2 monolayer was assessed using a trans-epithelial electrical resistance (TEER) probe (Merck Millipore, Billerica, USA), and cells were only used when resistance values were in excess of 600 ohms. During the assay, culture media was removed from wells and replaced with 15% FBS supplemented DMEM that had a final concentration of 10 μM aqueous solution or nanoemulsion equivalent of LPV or EFV. In different wells the apical or basolateral chambers (donor chambers) were loaded with drug or nanoemulsion containing media, with the opposite chambers (acceptor chambers) of those wells being filled with fresh drug-free media. Samples were taken from both apical and basolateral chambers from all wells

and immediately frozen at -40°C for subsequent batch analysis via HPLC. Sink conditions were determined as being satisfied when <10% of starting concentration had permeated. Papp was determined with the following equation:

$$Papp = \frac{\left(\frac{A}{T}\right) * (V)}{S * 0.3}$$

Where A is the concentration in the sample, T is the time in seconds at which the sample was collected, V is the total volume of sample contained in the chamber being sampled, S is the initial starting concentration of the drug and 0.3 is the surface area in cm2 of the transwell insert on which the Caco-2 cells are grown.

High Performance Liquid Chromatography

200 µL of sample (thawed from -40°C storage) was added to 1 ml of HPLC grade acetonitrile in a universal tube and vortexed for 30 seconds. The universal tubes where then centrifuged for 5 minutes at 4°C at 13,300 rpm, after which the supernatant was transferred to fresh glass tubes. These tubes were placed in a vacuum centrifuge drier and heated to 35°C to remove excess acetonitrile and dry down the sample to a pellet. Pellets were then re-suspended in 200 µL fresh 20% HPLC grade acetonitrile, 80% HPLC grade water, and transferred to 1.8 ml HPLC vials and sealed for HPLC analysis. The mobile phase (C) consisted of 95% water and 5% acetonitrile, with 5mM final concentration of ammonium formate. The elution buffer (D) consisted of 90% acetonitrile and 10% water with no ammonium formate. A calibration curve was produced using a 1:2 dilution from 20,000 ng/ml to 39 ng/ml, with separate quality control samples at 600 ng/ml, 3000 ng/ml and 15,000 ng/ml. The initial starting conditions of each run were 100% C, changing to 92% D / 8% C after 30 seconds, holding at this percentage for 5 minutes before raising to 100% D for 1 minute. The final 2 minutes of the run returned the column to 100% of C, ready for the injection of the next sample. The EFV was eluted at around 4.3 minutes into the run. The lower limit of quantification was 78 ng/mL, with intraday variability <20% and inter day variability <15%.

Antiviral Activity Assay

The lab adapted HIV-1 IIIB strain was added to MT4 cells in T-75 cell culture flasks containing 20 mL of pre-warmed RPMI cell culture media, supplemented with 10% sterile filtered FBS. HIV-1 IIIB replicates within the MT4 cells before budding out into the culture media. Viral particles were extracted and frozen at -80°C for future use. The multiplicity of infection (MOI) for the virus was 0.01. 1 vial of HIV-1 IIIB per 1 x 10^6 MT4 cells suspended in cell culture medium was defrosted and added to MT4 cells in a 50 ml falcon tube. This was centrifuged for 90 minutes at 5000 rpm and 4°C such that the viral particles were able to be in proximity to the MT4 cells and encourage infection of those cells. The supernatant was discarded and the cell-virus pellet re-suspended in fresh culture media to give a concentration of 20,000 cells per 80 µL. 80 µL of cell and virus suspension was plated into individual wells of a 96 well cell culture plate. To this, 20 µL of a 5x concentrated stock solution of candidate drug (aqueous solution or nanoemulsion formulations) was added, giving a final maximal concentration of 10 µM, reducing in a 1:1 dilution series to 0.00512 nM. Negative controls consisted of wells containing only cells and media, whereas positive controls consisted of wells containing cells, media and HIV-1 IIIB without the antiretroviral drug. Plates were incubated for 5 days at 37°C and 5% CO_2. Activity of both aqueous and nanoformulations against HIV-1 IIIB was determined by quantifying the amount of live cells in each condition by means of an MTT cell viability assay. Concentrations of LPV and EFV used were below that which showed cytotoxicity, ensuring that any observed cell death was due solely to HIV infection.

Statistical Analysis of Data

Statistical analysis was performed using SPSS version 21 for Mac, where data was normally distributed an independent samples t-test was performed to obtain p values. Where data was non-normally distributed a non-parametric Mann-Whitney U test was performed. P values are stated throughout. Sigmoidal dose response curves and subsequent CC50 values for cellular cytotoxicity were determined using GraphPad Prism Version 6 for Mac.

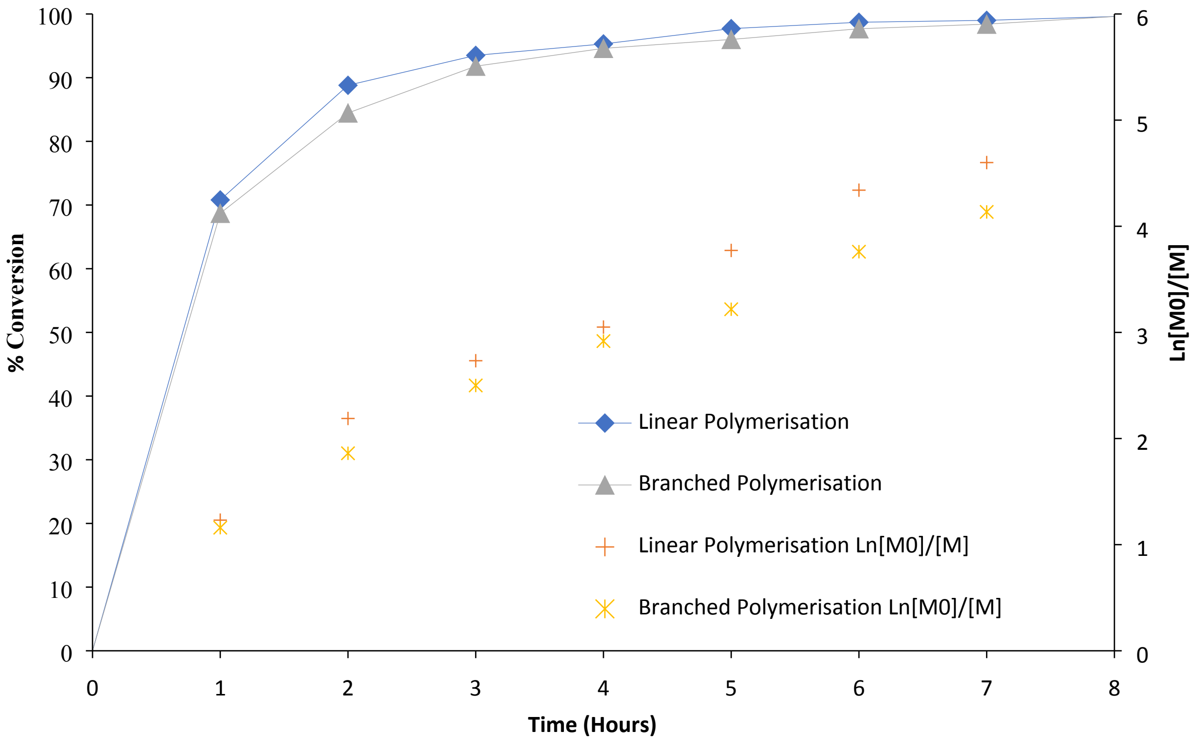

Figure S1. Kinetic studies of synthesis of DBiB*p*(OEGMA$_{90}$-*co*-EGDMA$_{0.95}$) *via* Cu-Catalysed ATRP in 92.5/7.5 IPA/$H_2$0 at 25 °C showing conversion and semilogarithmic plots *vs.* time.

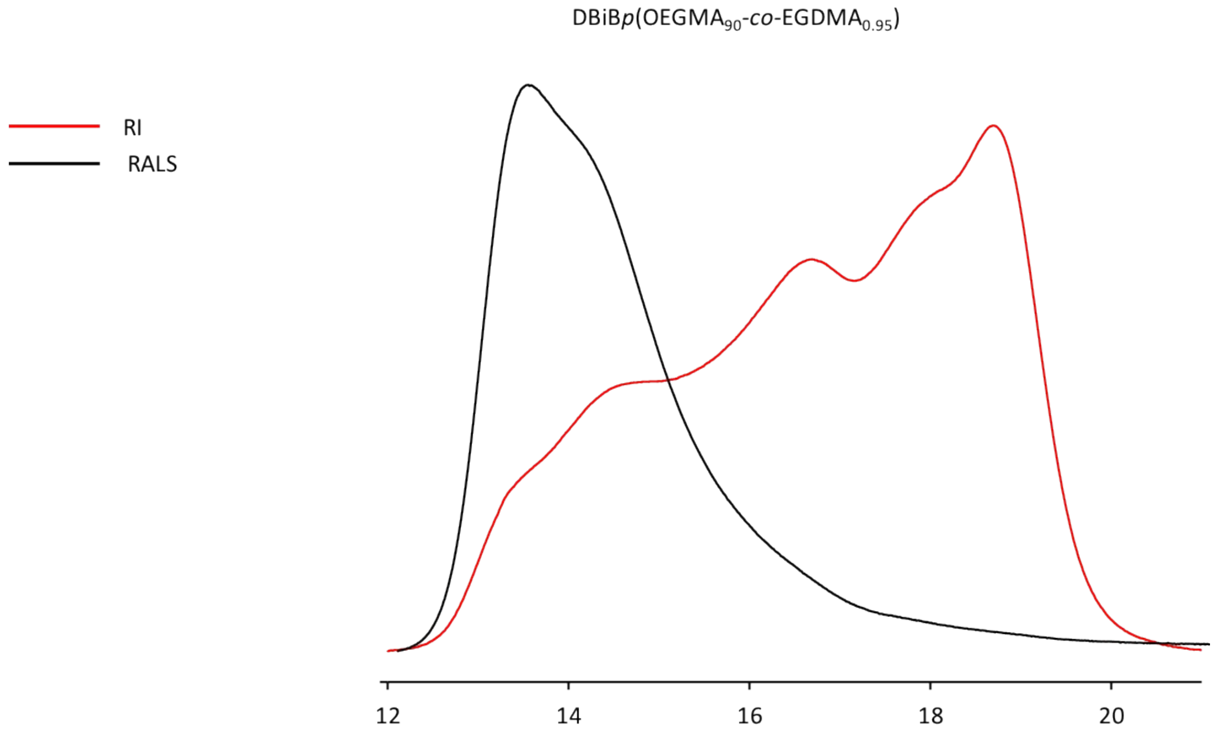

Figure S2. GPC trace of Branched polymer DBiB*p*(OEGMA$_{90}$-*co*-EGDMA$_{0.95}$) showing Refractive Index signal (red) and Right Angle Light Scattering signal (black)

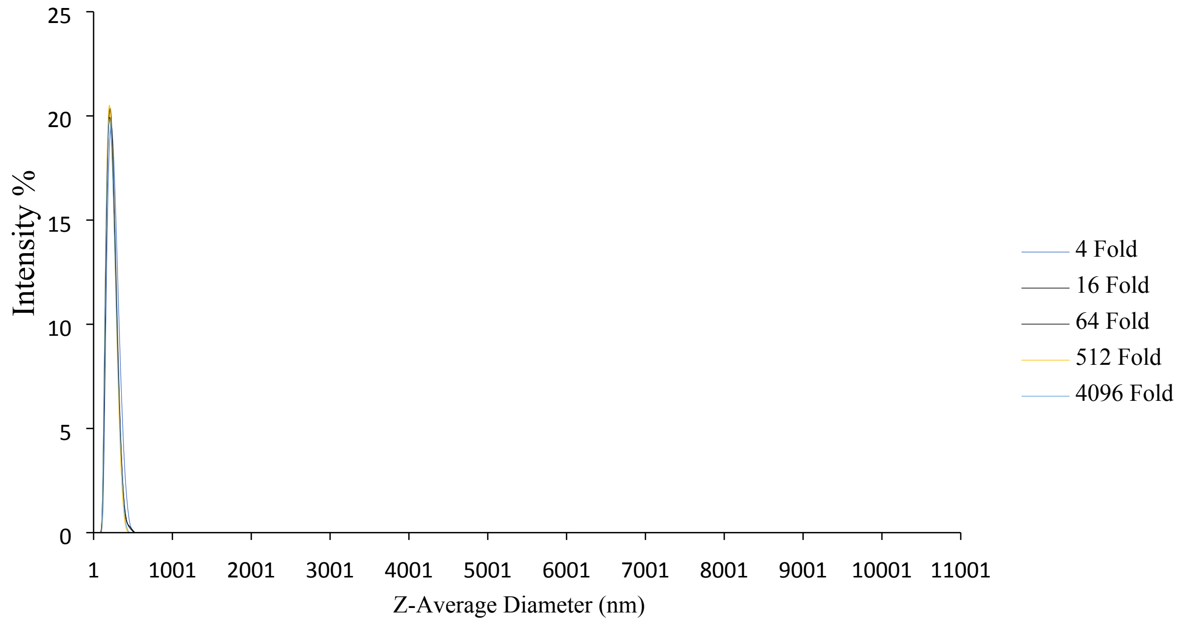

Figure S3. Stability of emulsion E65 when diluted in water up to 4096 fold, as determined by no change in Z-average diameters and size distributions. Data was obtained using dynamic light scattering (DLS) on a Malvern ZetaSizer Nano S.

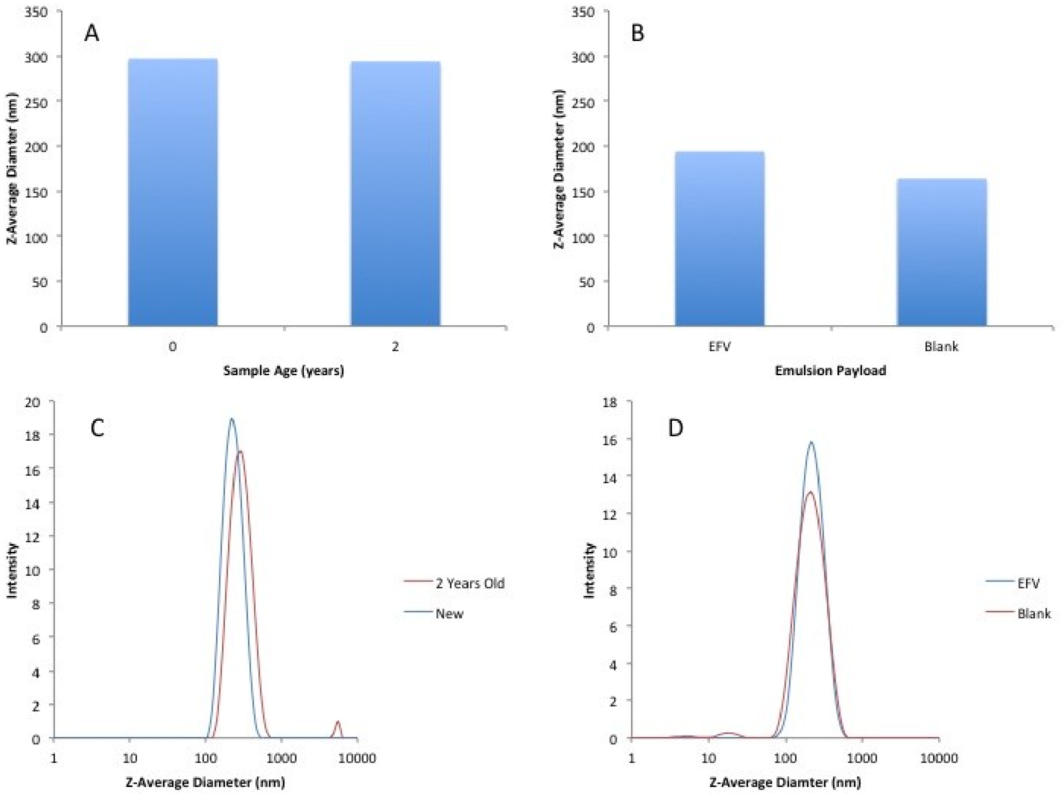

Figure S4. Two-year stability of emulsion E65 (A) and stability study of emulsion diluted in biological media after 3 months storage at 4°C (B). Corresponding DLS size distributions for two-year stability study (C) and media stability study (D). Data was obtained using dynamic light scattering, with a 100-fold dilution of neat emulsion sample in order to obtain laser attenuation between 5 and 7.

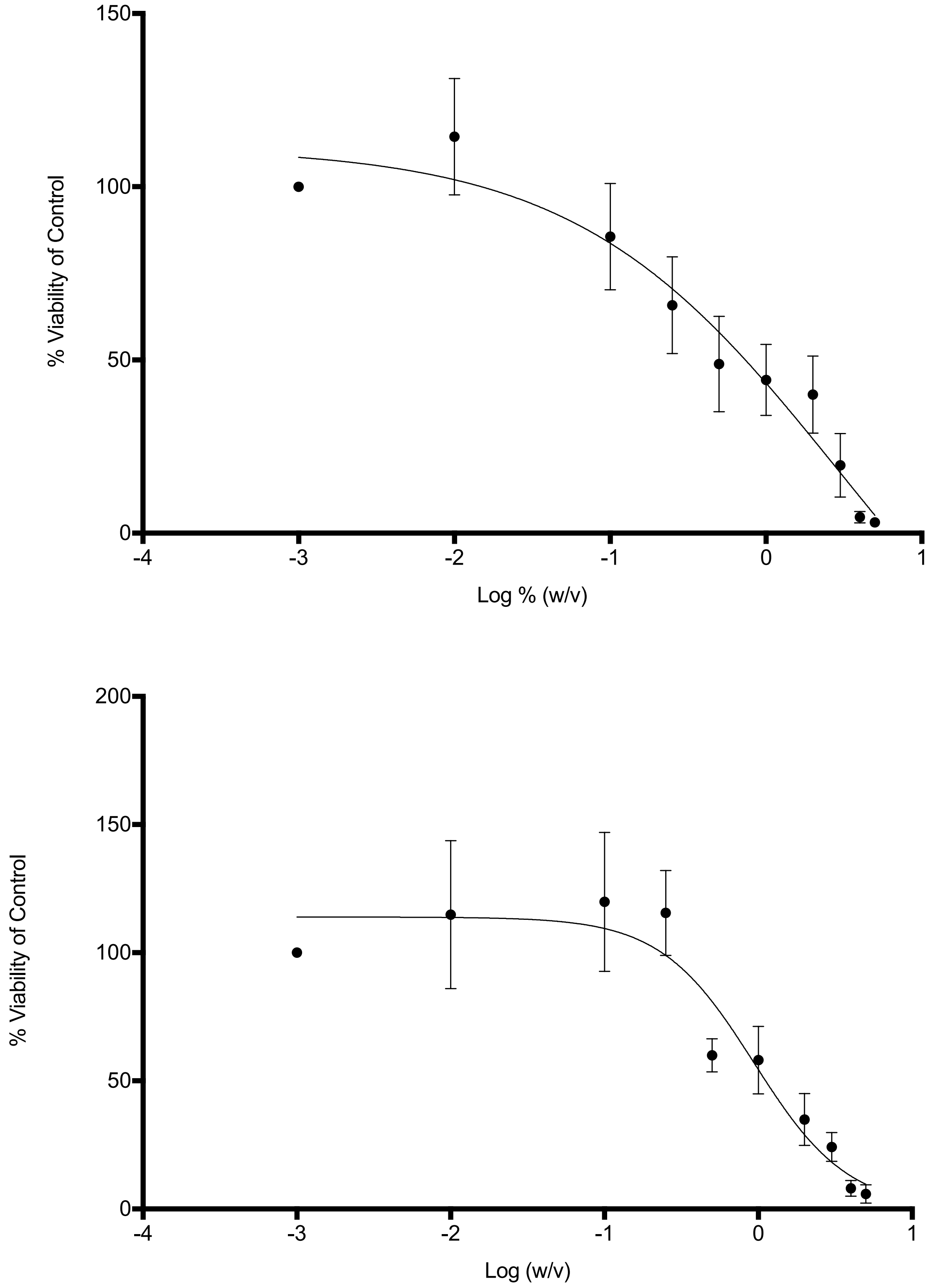

Figure S5. Effect of ATRP synthesised Branched PolyOEGMA DP_{80} on the metabolic activity of Caco-2 cells (top) and HepG2 cells (bottom) as determined by CellTiterGlo® ATP assay.

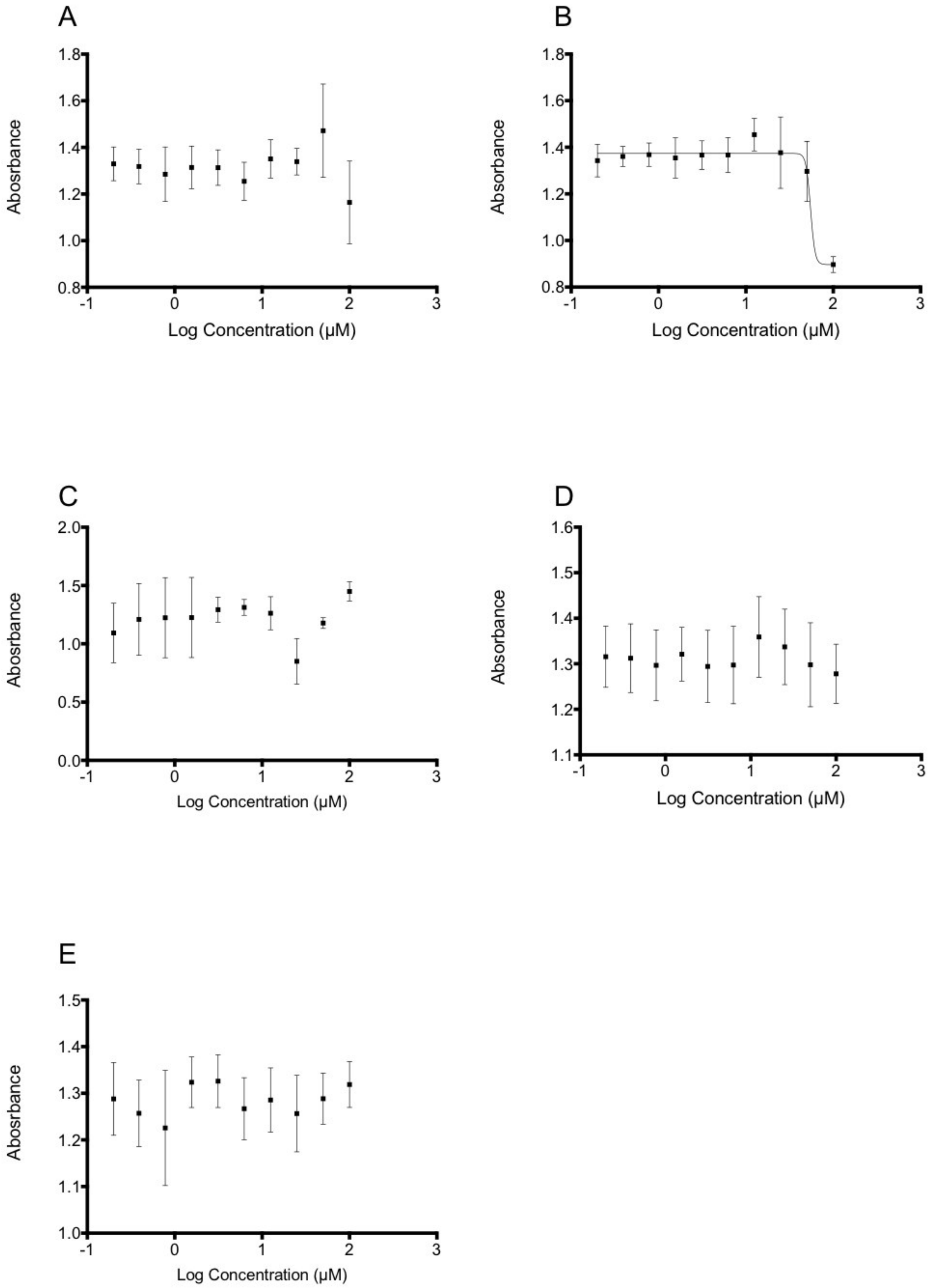

Figure S6. Effect of LPV aqueous solution (A), EFV aqueous solution (B), LPV nanoemulsion (C), EFV nanoemulsion (D) and blank nanoemulsion (E), on the metabolic activity of Caco-2 cells determined by MTT assay (Absorbance @ 560nm).

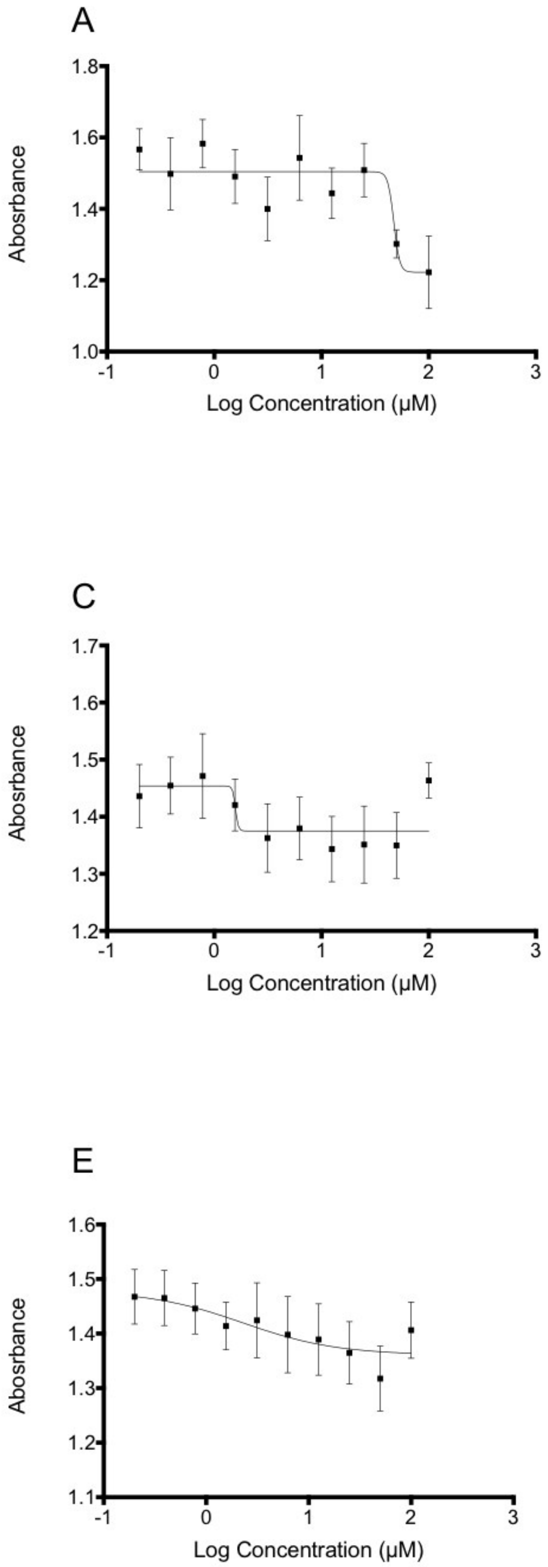

Figure S7. Effect of LPV aqueous solution (A), EFV aqueous solution (B), LPV nanoemulsion (C), EFV nanoemulsion (D) and blank nanoemulsion (E), on the metabolic activity of HepG-2 cells determined by MTT assay (Absorbance @560 nm).

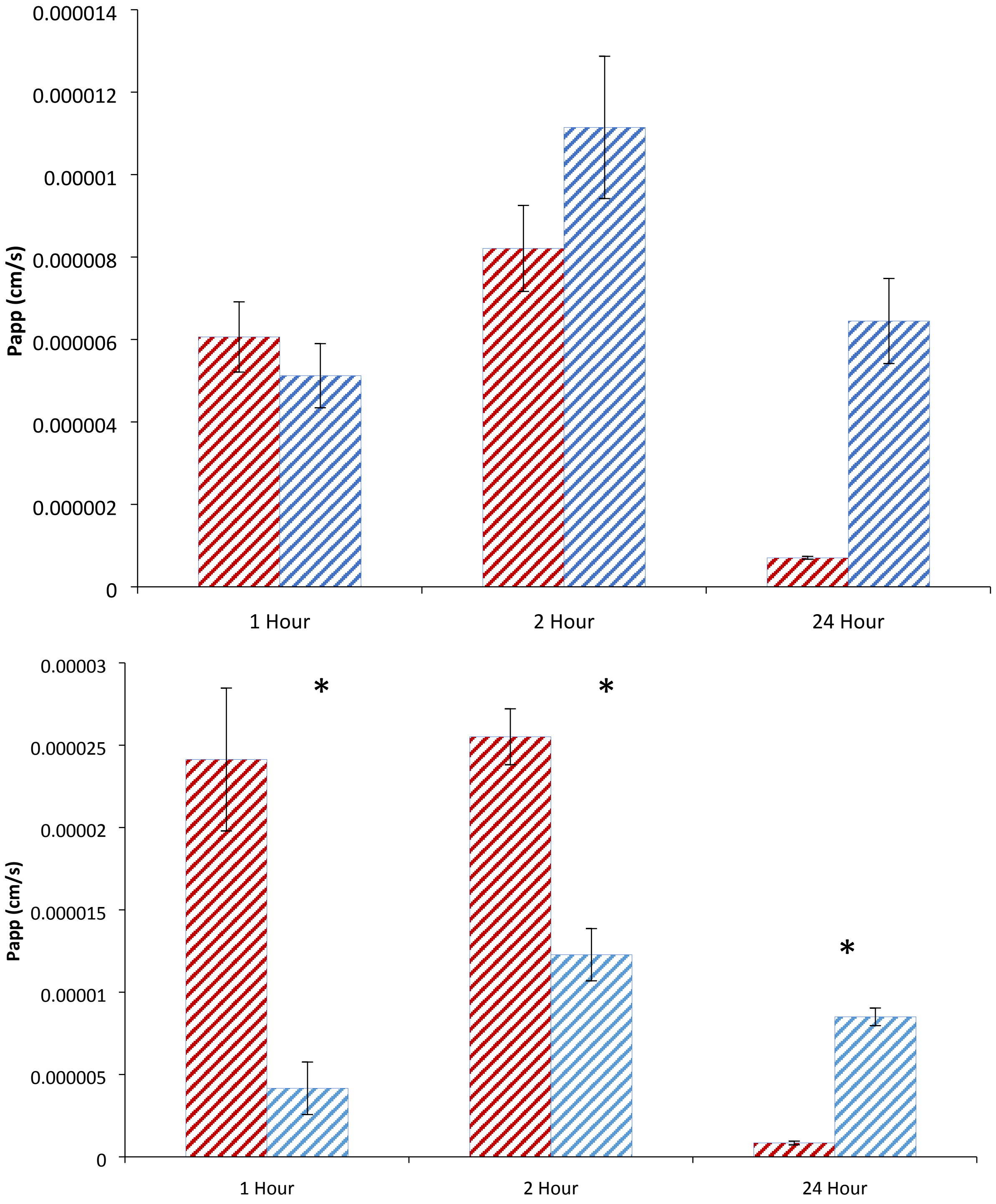

Figure S8. Apparent permeability of aqueous (red) and nanoemulsion (blue) formulations of EFV in the direction of apical to basolateral (top) and basolateral to apical (bottom). Samples were incubated for 1, 2 or 24 hours at a concentration of 10 µM EFV, added to the basolateral chamber. The full volume of media was taken from each chamber, for subsequent HPLC analysis. Data expressed as +/- standard deviation, N=4. *p=<0.05

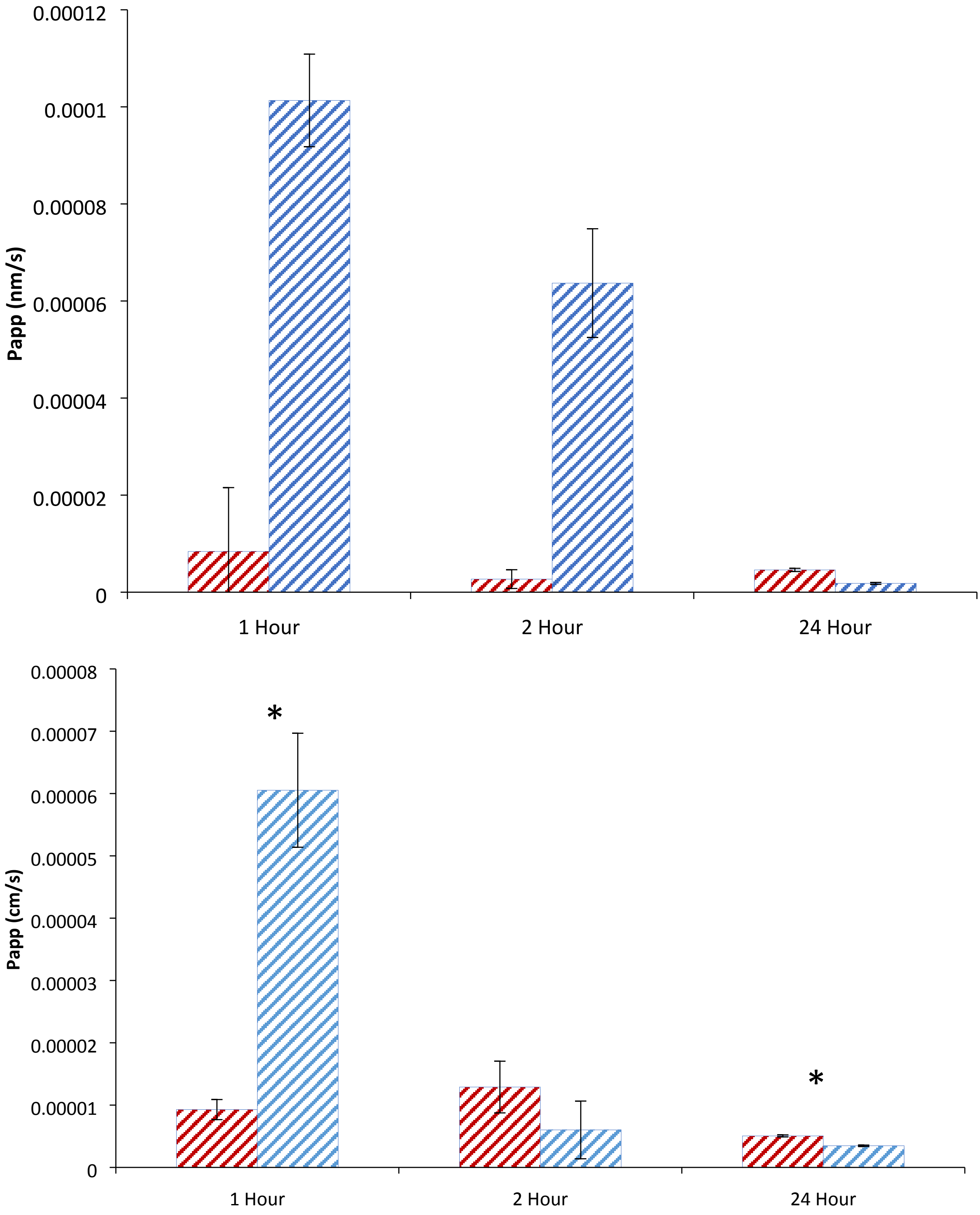

Figure S9. Apparent permeability of aqueous (red) and nanoemulsion (blue) formulations of LPV in the direction of basolateral to apical. Samples were incubated for 1, 2 or 24 hours at a concentration of 10 µM LPV, added to the basolateral chamber. The full volume of media was taken from each chamber, for subsequent HPLC analysis. Data expressed as +/- standard deviation, N=4. *p=<0.05

Table S1. Apparent permeation of efavirenz and lopinavir through Caco-2 monolayers when applied as either an aqueous DMSO solution or an aqueous nanoemulsion in castor oil. *All samples with the exception of nanoemulsion LPV satisfied sink conditions

	**EFV**<br>**A→B**<br>**(+/- SD)**	**p Value**	**B→A**<br>**(+/- SD)**	**p Value**
**1 Hour**				
Aqueous	6.06x10-6 (8.52x10-7)		2.41x10-5 (4.34x10-6)	
Nanoemulsion	5.12x1-6 (7.78x10-7)	> 0.05	4.16x10-6 (1.59x10-6)	< 0.05
**2 Hour**				
Aqueous	8.21x10-6 (1.04x10-6)		2.55x10-5 (1.70x10-6)	
Nanoemulsion	1.11x10-5 (1.73x10-6)	< 0.05	1.23x10-5 (1.59x10-6)	< 0.05
	**LPV**<br>**A→B**<br>**(+/- SD)**	**p Value**	**B→A**<br>**(+/- SD)**	**p Value**
**1 Hour**				
Aqueous	8.39x10-6 (1.32x10-5)		9.28x10-6 (1.61x10-6)	
Nanoemulsion	1.01x10-5 (9.54x10-6)	< 0.05	6.05x10-5 (9.15x10-6)	< 0.05
**2 Hour**				
Aqueous	2.70x10-6 (1.93x10-6)		1.29x10-5 (4.15x10-6)	
Nanoemulsion	6.37x10-5 (1.12x10-5)	< 0.05	6.02x10-6 (4.63x10-6)	> 0.05
